# Supplementary material for: Bayesian selection for coarse-grained models of liquid water
Source: Sci Rep. 2019 Jan 14;9:99. doi: 10.1038/s41598-018-37471-0 (PMC6331618; doi:10.1038/s41598-018-37471-0)
Supplement: Supplementary file 1 — Supporting Information for Bayesian selection for coarse-grained models of liquid water [file 41598_2018_37471_MOESM1_ESM.pdf]

Supporting Information for

Bayesian selection of coarse-grained models of liquid water

Julija Zavadlav, Georgios Arampatzis, and Petros Koumoutsakos*

Chair of Computational Science, ETH Zurich, Clausiusstrasse 33, CH-8092 Zurich, Switzerland

E-mail: petros@ethz.ch

1 Simulation details

In this section, we provide all the relevant information for the MD simulations and the Bayesian inference.

1.1 Molecular Dynamics

The MD simulations are performed with LAMMPS package.[1] For the calibration, we use a cubic box with periodic boundary conditions and 512 CG beads, corresponding to $512, 1024$, and 1536 particles for the $1, 2$, and 3 sited models. We use the Velocity Verlet integration with the time step of 2 fs. In rigid models, the bonds/angles are constrained with RATTLE. The cut-off radius for the nonbonded interactions is 3σ, where σ is the free parameter of the LJ interaction. The electrostatic interactions beyond the cut-off are corrected with the particle-particle particle-mesh solver scheme. The temperature and pressure of 1 atm (for the NpT) are maintained with Nose-Hover with time constants of $\tau_T = 0.1$ ps and $\tau_p = 1.0$ ps,

respectively. Density ρ is evaluated in the NpT ensemble. For the surface tension γ of the water/vapor interface, the water is surrounded by two vacuum regions in the z direction. It is computed in the NVT ensemble as $\gamma = L_z/2\langle p_{zz} - p_{||}\rangle$, where where L_z is the box length in the z-direction and the p_z and $p_{||}$ denote the perpendicular and the lateral pressure components. The dielectric constant ε is computed from the fluctuations of the total dipole moment, while the shear viscosity η is calculated according to the Green-Kubo formula as $\eta = V/(3k_bT)\int_0^\infty \sum_{a<b} p_{ab}(t)p_{ab}(0)dt$, where V is the system volume, k_B is the Boltzmann constant, and p_{ab} are the off-diagonal components of the pressure tensor ($\alpha\beta = xy, xz, yz$) in the NVT ensemble. The isothermal compressibility κ is evaluated under the NpT conditions as $\kappa = (\langle V^2\rangle - \langle V\rangle^2)/(k_bTV)$.

For the Bayesian inference, the protocol for the simulations is the following: a 100 ps equilibration in the NVT ensemble followed by 100 ps equilibration in the NpT ensemble followed by 1 ns production run. For the evaluation of γ, the system is additionally equilibrated for 100 ps under NVT conditions. Additional evaluations of each model's properties are performed for the parameters with the maximum likelihood. We first evaluate the maximum integration time-step Δt by performing a series of NVE simulations varying the Δt and monitoring the conservation of the total energy. Using the maximum Δt, approximately doubled size of the system, 1 ns equilibration, and 10 ns production runs we evaluate the properties $\rho, \varepsilon, \gamma, \kappa$ as stated above.

1.2 Uncertainty Quantification

In most practical applications, the posterior distribution

$$p(\boldsymbol{\varphi}|\,\boldsymbol{d}, \mathcal{M}) = \frac{p(\boldsymbol{d}|\,\boldsymbol{\varphi}, \mathcal{M})\; p(\boldsymbol{\varphi}|\mathcal{M})}{p(\boldsymbol{d}|\,\mathcal{M})}\,, \tag{S1}$$

can not be expressed analytically. Moreover, the normalizing constant $p(\boldsymbol{d}|\mathcal{M})$ is not known since it is given by integration of the denominator in Eq. (S1) over the potentially high

dimensional domain of the parameters. Hence, we rely on efficient sampling algorithms to identify the posterior distribution. In the present work, we use the Transitional Markov Chain Monte Carlo algorithm (TMCMC).[2] The algorithm starts by sampling a *population* from the prior distribution, which usually is trivial to be sampled, and through annealing steps provides samples from the posterior distribution. Three major advantages of this algorithm are its ability to sample multimodal distributions, in cases where single chain MCMC methods may fail, the algorithm provides an asymptotically unbiased estimator of the evidence[3] (see SI), and the fact that it can run in parallel. We report that in all simulations the parameter β^2 was set to 0.04.

The parallel property of the algorithm is very important in this study since the model evaluation can take on the order of an hour for execution. An efficient implementation of TMCMC can be found in Π4U[1], a platform agnostic high performance framework for uncertainty quantification. The sampling algorithm is further accelerated by using surrogate models based on Gaussian processes (see section 1.3) leading to a 10-20% speedup.

The data on which the Bayesian uncertainty quantification is performed are experimental data for density, dielectric constant (not used in the LJ model as it is chargeless), surface tension of the water/vapor interface, isothermal compressibility, and shear viscosity (see SI). We want to allow different levels of error for different data thus we choose the covariance matrix Σ in the main text to be equal to,

$$\Sigma = \sigma_n^2 \, \mathrm{diag} \left(d_1, 4d_2, 8d_3, 8d_4, 8d_5 \right)^2 \ . \tag{S2}$$

In order to select the best model, we work as follows. First, we perform the inference on all the parameters of each model using all five QoI. Next, we fix all parameters except σ and ϵ of the LJ interaction to the values of the maximum a posterior estimation, i.e., the parameter

[1] Π4U can be downloaded from here: https://github.com/cselab/pi4u

with the highest probability,

$$\boldsymbol{\varphi}^\star = \arg\max_{\boldsymbol{\varphi}} p(\boldsymbol{\varphi} | \boldsymbol{d}) \ . \tag{S3}$$

Finally, we infer the remaining parameters of the models excluding the dielectric constant from the QoI.

Our motivation for this approach is three-fold. First, we remove the dependency of the evidence on the number of model parameters. The complexity of the model is then measured through the computational cost of the model. This measure is more appropriate as two models can have the same number of the parameters but could differ in the computational cost. Second, the evidence of the models can be directly compared to the evidence of the LJ model. Finally, having only 3 parameters reduces the demand on the population size, thus reducing the computational cost of the sampling algorithm.

1.3 Surrogate models

The computational cost of the Bayesian UQ lies in the computation of the likelihood term in Eq. S1. Each likelihood evaluation implies a model evaluation, i.e., a computational demanding simulation using LAMMPS. The idea of *surrogate models*[4] is to use special interpolation techniques in order to substitute the heavy model evaluations.

In this work we choose to use Gaussian Processes[5] (GPs) as a surrogate for the likelihood. Following the notation in Bishop,[5] given the observed data $\boldsymbol{t} = (t_1, \ldots, t_N)$ that correspond to input $\boldsymbol{x} = (x_1, \ldots, x_N)$ the conditional distribution $p(t_{N+1}|\boldsymbol{t})$ is given by

$$p(t_{N+1}|\boldsymbol{t}) = \mathcal{N}(t_{N+1} \,|\, m(x_{N+1}), \sigma^2(x_{N+1})) \ , \tag{S4}$$

and

$$m(x_{N+1}) = \boldsymbol{k}^T \boldsymbol{C}_N^{-1} \boldsymbol{t} \quad \text{and} \quad \sigma^2(x_{N+1}) = c - \boldsymbol{k}^T \boldsymbol{C}_N^{-1} \boldsymbol{k} \ , \tag{S5}$$

where $\boldsymbol{k}_i = k(x_i, x_{N+1})$, $i = 1, \ldots, N$, $c = k(x_{N+1}, x_{N+1}) + \beta^{-1}$ and the i, j element of the matrix $\boldsymbol{C}_N$ is given by $k(x_i, x_j) + \beta^{-1}\delta_{ij}$ for $i, j = 1, \ldots, N$. The scalar β is the error assumed

on the observed data. The kernel function $k(x, y)$ models the fact that points which are close in the input space are expected to have more strongly correlated outputs. In general, the kernel function depends on hyper-parameters which are learned through an optimization process. In this work, we use the squared exponential kernel.

In the context of TMCMC, the training set $\boldsymbol{t}_N$ consists of likelihoods values t_i computed on parameters x_i based only on the exact model evaluation. Based on these values, a GP model is trained and evaluated on a new point (t_{N+1}, x_{N+1}). If the relative error $\sigma(x_{N+1})/|m(x_{N+1})|$ is less than a threshold then the likelihood value at x_{N+1} is set to $m(x_{N+1})$, otherwise, the model is computed and the likelihood is evaluated. Here, we choose the threshold to be 0.05.

In order to make more accurate surrogate models, we choose to train a GP model on a subdomain of the finite initial domain centered on x_{N+1}. Here, we choose to train on 25% of the initial domain.

2 Hierarchical UQ

We follow the methodology developed in Ref.[6] We assume that data comes split in N different datasets: $\vec{\boldsymbol{d}} = \{\boldsymbol{d}_1, \dots, \boldsymbol{d}_N\}$.

The likelihood in the probabilistic model $\mathcal{M}_i$ is $\mathcal{N}(\boldsymbol{d}_i \,|\, f(\boldsymbol{x}_i; \boldsymbol{\varphi}_i), \boldsymbol{\Sigma}_i)$. We assume that the probability of $\boldsymbol{\varphi}_i$ depends on a hyper-parameter $\boldsymbol{\psi} \in \mathbb{R}^{N_\psi}$ and is given by a PDF $p(\boldsymbol{\varphi} \,|\, \boldsymbol{\psi}, \mathcal{M})$, where $\mathcal{M}$ corresponds to the graph describing the relations between $\boldsymbol{\psi}$, $\boldsymbol{\varphi}_i$ and $\boldsymbol{d}_i$, see Figure S1. Our goal is to obtain samples from the posterior distribution, $p(\boldsymbol{\varphi}_i \,|\, \vec{\boldsymbol{d}}, \mathcal{M})$,

$$p(\boldsymbol{\varphi}_i \,|\, \vec{\boldsymbol{d}}, \mathcal{M}) = \int p(\boldsymbol{\varphi}_i \,|\, \boldsymbol{\psi}, \vec{\boldsymbol{d}}, \mathcal{M})\, p(\boldsymbol{\psi} \,|\, \vec{\boldsymbol{d}}, \mathcal{M})\, d\boldsymbol{\psi}\,. \tag{S6}$$

where $\vec{\boldsymbol{d}} = \{\boldsymbol{d}_1, \dots, \boldsymbol{d}_N\}$. The dependency assumptions from Fig. S1 allow to simplify: $p(\boldsymbol{\varphi}_i \,|\, \boldsymbol{\psi}, \vec{\boldsymbol{d}}, \mathcal{M}) = p(\boldsymbol{\varphi}_i \,|\, \boldsymbol{\psi}, \boldsymbol{d}_i, \mathcal{M})$, and equation (S6) can be rewritten using the Bayes'

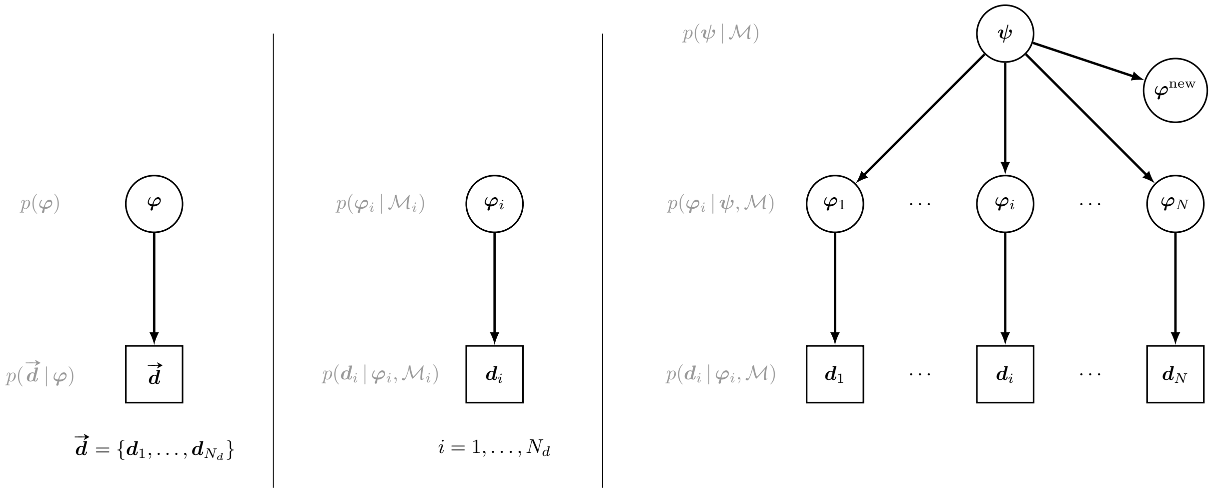

Figure S1: Bayesian networks: (left) network for the i-th dataset, (right) hierarchical Bayesian network.

theorem:

$$p(\boldsymbol{\varphi}_i\,|\,\vec{\boldsymbol{d}}, \mathcal{M}) = \int \frac{p(\boldsymbol{d}_i\,|\,\boldsymbol{\varphi}_i, \boldsymbol{\psi}, \mathcal{M})\, p(\boldsymbol{\varphi}_i\,|\,\boldsymbol{\psi}, \mathcal{M})}{p(\boldsymbol{d}_i\,|\,\boldsymbol{\psi}, \mathcal{M})}\, p(\boldsymbol{\psi}\,|\,\vec{\boldsymbol{d}}, \mathcal{M})\, d\boldsymbol{\psi}\,. \tag{S7}$$

Since $p(\boldsymbol{d}_i\,|\,\boldsymbol{\varphi}_i, \boldsymbol{\psi}, \mathcal{M}) = p(\boldsymbol{d}_i\,|\,\boldsymbol{\varphi}_i, \mathcal{M})$, equation (S7) simplifies to

$$p(\boldsymbol{\varphi}_i\,|\,\vec{\boldsymbol{d}}, \mathcal{M}) = \quad p(\boldsymbol{d}_i\,|\,\boldsymbol{\varphi}_i, \mathcal{M}) \int \frac{p(\boldsymbol{\varphi}_i\,|\,\boldsymbol{\psi}, \mathcal{M})}{p(\boldsymbol{d}_i\,|\,\boldsymbol{\psi}, \mathcal{M})}\, p(\boldsymbol{\psi}\,|\,\vec{\boldsymbol{d}}, \mathcal{M})\, d\boldsymbol{\psi}\,. \tag{S8}$$

Finally, the posterior distribution (S6) can be approximated as

$$p(\boldsymbol{\varphi}_i\,|\,\vec{\boldsymbol{d}}, \mathcal{M}) \approx \frac{p(\boldsymbol{d}_i\,|\,\boldsymbol{\varphi}_i, \mathcal{M})}{N_s} \sum_{k=1}^{N_s} \frac{p(\boldsymbol{\varphi}_i\,|\,\boldsymbol{\psi}^{(k)}, \mathcal{M})}{p(\boldsymbol{d}_i\,|\,\boldsymbol{\psi}^{(k)}, \mathcal{M})}\,, \tag{S9}$$

where, $\boldsymbol{\psi}^{(k)} \sim p(\boldsymbol{\psi}\,|\,\vec{\boldsymbol{d}}, \mathcal{M})$ and N_s is sufficiently large. Thus, in order to obtain $\boldsymbol{\varphi}_i$ samples, we first have to sample the probability distribution $p(\boldsymbol{\psi}\,|\,\vec{\boldsymbol{d}}, \mathcal{M})$, which, according to Bayes' theorem, is equal to

$$p(\boldsymbol{\psi}\,|\,\vec{\boldsymbol{d}}, \mathcal{M}) = \frac{p(\vec{\boldsymbol{d}}\,|\,\boldsymbol{\psi}, \mathcal{M})\, p(\boldsymbol{\psi}\,|\,\mathcal{M})}{p(\vec{\boldsymbol{d}}\,|\,\mathcal{M})}\,, \tag{S10}$$

where $p(\boldsymbol{\psi}\,|\,\mathcal{M})$ is the prior PDF on $\boldsymbol{\psi}$ and $p(\vec{\boldsymbol{d}}\,|\,\mathcal{M})$ is the normalizing constant. Exploiting

the dependency assumption of Fig. S1 we see that

$$p(\vec{\boldsymbol{d}}\,|\,\boldsymbol{\psi},\mathcal{M}) = \prod_{i=1}^{N} p(\boldsymbol{d}_i\,|\,\boldsymbol{\psi},\mathcal{M})\,, \tag{S11}$$

and the likelihood of i-th dataset can be expressed according to the total probability theorem as

$$p(\boldsymbol{d}_i\,|\,\boldsymbol{\psi},\mathcal{M}) = \int p(\boldsymbol{d}_i\,|\,\boldsymbol{\varphi}_i,\mathcal{M})\, p(\boldsymbol{\varphi}_i\,|\,\boldsymbol{\psi},\mathcal{M})\, d\boldsymbol{\varphi}_i\,. \tag{S12}$$

Here we introduce the model $\mathcal{M}_i$ described in Fig. S1. The posterior distribution of this model will be used as instrumental density for important sampling. Under the modeling assumption $p(\boldsymbol{d}_i\,|\,\boldsymbol{\varphi}_i,\mathcal{M}) = p(\boldsymbol{d}_i\,|\,\boldsymbol{\varphi}_i,\mathcal{M}_i)$ (see[6]) and the use of the Bayes' theorem, equation (S12) is written as

$$p(\boldsymbol{d}_i\,|\,\boldsymbol{\psi},\mathcal{M}) = \int \frac{p(\boldsymbol{\varphi}_i\,|\,\boldsymbol{d}_i,\mathcal{M}_i)\, p(\boldsymbol{d}_i\,|\,\mathcal{M}_i)}{p(\boldsymbol{\varphi}_i\,|\,\mathcal{M}_i)}\, p(\boldsymbol{\varphi}_i\,|\,\boldsymbol{\psi},\mathcal{M})\, d\boldsymbol{\varphi}_i\,, \tag{S13}$$

or, equivalently, as

$$p(\boldsymbol{d}_i\,|\,\boldsymbol{\psi},\mathcal{M}) = p(\boldsymbol{d}_i\,|\,\mathcal{M}_i) \int \frac{p(\boldsymbol{\varphi}_i\,|\,\boldsymbol{\psi},\mathcal{M})}{p(\boldsymbol{\varphi}_i\,|\,\mathcal{M}_i)}\, p(\boldsymbol{\varphi}_i\,|\,\boldsymbol{d}_i,\mathcal{M}_i)\, d\boldsymbol{\varphi}_i\,. \tag{S14}$$

Finally, equation (S12) can be approximated as

$$p(\boldsymbol{d}_i\,|\,\boldsymbol{\psi},\mathcal{M}) \approx \frac{p(\boldsymbol{d}_i\,|\,\mathcal{M}_i)}{N_s} \sum_{k=1}^{N_s} \frac{p(\boldsymbol{\varphi}_i^{(k)}\,|\,\boldsymbol{\psi},\mathcal{M})}{p(\boldsymbol{\varphi}_i^{(k)}\,|\,\mathcal{M}_i)}\,, \tag{S15}$$

where $\boldsymbol{\varphi}_i^{(k)} \sim p(\boldsymbol{\varphi}_i\,|\,\boldsymbol{d}_i,\mathcal{M}_i)$ and N_s is sufficiently large. Note that in general N_s can be different for each data set $\boldsymbol{d}_i$. The advantage of this approach is that the likelihoods $p(\boldsymbol{d}_i\,|\,\boldsymbol{\varphi}_i,\mathcal{M}_i)$, $i = 1,\ldots,N$, which are the most expensive part of the computations, are not re-evaluated for each $\boldsymbol{\psi}$.

3 Data

Table S1: Experimental data[7,8] for density ρ, dielectric constant ε, surface tension γ, isothermal compressibility κ, and shear viscosity η used as target objective.

T [°]	T [K]	ρ [g/cm^3]	ε [ε_0]	γ [mN/m]	κ [10^{-6}/bar]	η [mPa s]
10	283	0.999	84.0	74.2	47.89	1.307
25	298	0.997	78.4	72.0	45.24	0.891
50	323	0.988	69.9	67.9	44.17	0.547

Table S2: Properties of reference water models at $T = 298$ K used for comparison.

model	exp[7,8]	MARTINI[9,10]	MARTINI Pol.[10]	BMW[11,12]	GROMOS CG[13]
ρ [g/cm^3]	0.997	0.99	1.043	1.047	0.995
ε [ε_0]	78.4	/	75.6	74	73.7
γ [mN/m]	72.0	30-40	30.5	77	51.2
κ [10^{-6}/bar]	45.24	60.0	/	33	84-138
η [mPa s]	0.891	/	/	1.01-1.62	3.72

4 UQ inference for density, dielectric constant, surface tension, isothermal compressibility, and shear viscosity

A uniform prior distribution is used for all model parameters. The population size of the TMCMC is 3000 for the *2S* model. For the *2SF 3S*, and *3S** model it is 4000, while for the *3SF* and *3S*F* models it is 5000.

Table S3: Model parameter ranges (σ in Å, ϵ in kcal/mol, e in e_0, l in Å, and θ in °) for the considered rigid models and mappings M. The dipole moment μ is given in D.

2S M	σ	ϵ	e	l	σ	μ
3	4.15 - 4.55	0.30 - 1.00	0.30 - 1.01	1.1 - 2.8	0.0 - 0.1	4.4 - 5.9
4	4.60 - 5.00	0.30 - 1.00	0.30 - 1.01	1.1 - 2.8	0.0 - 0.1	5.1 - 6.6
5	5.00 - 5.40	0.30 - 1.00	0.30 - 1.01	1.1 - 2.8	0.0 - 0.1	5.4 - 6.9
6	5.30 - 5.70	0.30 - 1.00	0.30 - 1.01	1.1 - 2.8	0.0 - 0.1	5.8 - 7.3
12	6.50 - 7.10	0.30 - 1.15	0.30 - 1.01	1.7 - 3.2	0.0 - 0.1	8.0 - 11.0

3S M	σ	ϵ	e	l	θ	σ	μ
3	4.15 - 4.55	0.30 - 1.00	0.30 - 1.01	1.1 - 2.8	0 - 140	0.0 - 0.1	4.4 - 5.9
4	4.60 - 5.00	0.30 - 1.00	0.30 - 1.01	1.1 - 2.8	0 - 140	0.0 - 0.1	5.1 - 6.6
5	5.00 - 5.40	0.30 - 1.00	0.30 - 1.01	1.1 - 2.8	0 - 140	0.0 - 0.1	5.4 - 6.9
6	5.30 - 5.70	0.30 - 1.00	0.30 - 1.01	1.1 - 2.8	0 - 140	0.0 - 0.1	5.8 - 7.3
12	6.50 - 7.10	0.30 - 1.15	0.30 - 1.01	1.7 - 3.2	0 - 140	0.0 - 0.1	8.0 - 11.0

*3S** M	σ	ϵ	e	l	θ	σ	μ
3	4.15 - 4.55	0.30 - 1.00	0.30 - 1.01	0.8 - 1.5	40 - 180	0.0 - 0.1	4.4 - 5.9
4	4.60 - 5.00	0.30 - 1.00	0.30 - 1.01	0.8 - 1.5	40 - 180	0.0 - 0.1	5.1 - 6.6
5	5.00 - 5.40	0.30 - 1.00	0.30 - 1.01	0.8 - 1.5	40 - 180	0.0 - 0.1	5.4 - 6.9
6	5.30 - 5.70	0.30 - 1.00	0.30 - 1.01	0.8 - 1.5	40 - 180	0.0 - 0.1	5.8 - 7.3
12	6.70 - 7.30	0.30 - 1.15	0.30 - 1.01	1.2 - 2.0	40 - 180	0.0 - 0.1	8.0 - 11.0

Table S4: Model parameter ranges (σ in Å, ϵ in kcal/mol, e in e_0, l in Å, k_B in kcal/mol/nm^2, θ in °, and k_A in kcal/mol) for the considered flexible models and mappings $M = 4$. The dipole moment μ is given in D.

model	σ	ϵ	e	l	k_B	μ
2SF	4.60 - 5.00	0.30 - 1.00	0.30 - 1.01	1.1 - 2.8	10 - 60	5.1 - 6.6

model	σ	ϵ	e	l	θ	k_A	σ	μ
3SF	4.60 - 5.00	0.30 - 1.00	0.30 - 1.01	1.1 - 2.8	0 - 140	1 - 10	0.0 - 0.1	5.1 - 6.6

model	σ	ϵ	e	l	θ	k_A	σ	μ
*3S*F*	4.60 - 5.00	0.30 - 1.00	0.30 - 1.01	0.8 - 1.5	40 - 180	1 - 10	0.0 - 0.1	5.1 - 6.6

Table S5: Model parameters (σ in Å, ϵ in kcal/mol, e in e_0, l in Å, k_B in kcal/mol/nm^2, θ in °, and k_A in kcal/mol) with maximum likelihood for the considered rigid models and mappings M. The dipole moment μ is given in D.

2S					
M	σ	ϵ	e	l	μ
3	4.3912	0.9699	0.6213	1.5056	4.4934
4	4.8491	0.9690	0.6596	1.6880	5.3478
5	5.2222	0.9982	0.7197	1.6171	5.5908
6	5.5018	0.9928	0.8683	1.3967	5.8255
12	6.9531	0.9662	0.7707	2.5069	9.2808

3S						
M	σ	ϵ	e	l	θ	μ
3	4.4119	0.9512	0.6047	1.6138	3.5880	4.6850
4	4.8468	0.8782	0.6285	1.8062	7.1728	5.4420
5	5.2159	0.9926	0.7262	1.6778	6.9914	5.8413
6	5.5299	0.9808	0.6428	2.1903	9.0216	6.7420
12	6.9527	1.0053	0.8351	2.3593	53.9798	8.4340

*3S**						
M	σ	ϵ	e	l	θ	μ
3	4.4040	0.8625	1.0053	0.9551	57.4459	4.4310
4	4.8898	0.9872	0.8219	1.2458	65.4964	5.3182
5	5.2212	0.9654	0.9112	1.1455	66.0899	5.4656
6	5.5341	0.9943	0.9143	1.2926	62.9153	5.9224
12	7.0897	0.9186	0.8780	1.8466	86.7040	10.6878

Table S6: Model parameters (σ in Å, ϵ in kcal/mol, e in e_0, l in Å, k_B in kcal/mol/nm^2, θ in °, and k_A in kcal/mol) with maximum likelihood for the considered flexible models and mappings $M = 4$. The dipole moment μ is given in D.

model	σ	ϵ	e	l	k_B	μ
2SF	4.9172	0.9396	0.5452	2.4337	34.8621	6.3730

model	σ	ϵ	e	l	θ	k_A	μ
3SF	4.8348	0.9395	0.6493	1.7219	29.3119	9.2364	5.1953
model	σ	ϵ	e	l	θ	k_A	μ
*3S*F*	4.8982	0.9092	0.5495	1.4730	87.0474	7.8318	5.3524

5 UQ inference for density, surface tension, isothermal compressibility, and shear viscosity

A uniform prior distribution is used for all model parameters. The population size of the TMCMC is 2000 for all models.

Table S7: Model parameter ranges (σ in Å, ϵ in kcal/mol, e in e_0, l in Å, and θ in °) for the considered rigid models and mappings M. The dipole moment μ is given in D.

LJ			
M	σ	ϵ	σ
1	2.80 - 3.00	0.50 - 1.25	0.0 - 0.1
3	4.00 - 4.40	0.50 - 1.25	0.0 - 0.1
4	4.45 - 4.85	0.50 - 1.25	0.0 - 0.1
5	4.80 - 5.30	0.50 - 1.25	0.0 - 0.1
6	5.10 - 5.60	0.50 - 1.25	0.0 - 0.1

2S					fixed	
M	σ	ϵ	σ	e	l	μ
3	4.15 - 4.55	0.30 - 1.15	0.0 - 0.1	0.6213	1.5056	4.4934
4	4.60 - 5.00	0.30 - 1.15	0.0 - 0.1	0.6596	1.6880	5.3478
5	5.00 - 5.40	0.30 - 1.15	0.0 - 0.1	0.7197	1.6171	5.5908
6	5.30 - 5.70	0.30 - 1.15	0.0 - 0.1	0.8683	1.3967	5.8255
12	6.50 - 7.10	0.30 - 1.15	0.0 - 0.1	0.7707	2.5069	9.2808

3S					fixed		
M	σ	ϵ	σ	e	l	θ	μ
3	4.15 - 4.55	0.30 - 1.15	0.0 - 0.1	0.6047	1.6138	3.5880	4.685
4	4.60 - 5.00	0.30 - 1.15	0.0 - 0.1	0.6285	1.8062	7.1728	5.4420
5	5.00 - 5.40	0.30 - 1.15	0.0 - 0.1	0.7262	1.6778	6.9914	5.8413
6	5.30 - 5.70	0.30 - 1.15	0.0 - 0.1	0.6428	2.1903	9.0216	6.7420
12	6.50 - 7.10	0.30 - 1.15	0.0 - 0.1	0.8351	2.3593	53.9798	8.4340

*3S**					fixed		
M	σ	ϵ	σ	e	l	θ	μ
3	4.15 - 4.55	0.30 - 1.15	0.0 - 0.1	1.0053	0.9551	57.4459	4.4310
4	4.60 - 5.00	0.30 - 1.15	0.0 - 0.1	0.8219	1.2458	65.4964	5.3182
5	5.00 - 5.40	0.30 - 1.15	0.0 - 0.1	0.9112	1.1455	66.0899	5.4656
6	5.30 - 5.70	0.30 - 1.15	0.0 - 0.1	0.9143	1.2926	62.9153	5.9224
12	6.70 - 7.30	0.30 - 1.15	0.0 - 0.1	0.8780	1.8466	86.7040	10.6878

Table S8: Model parameter ranges (σ in Å, ϵ in kcal/mol, e in e_0, l in Å, k_B in kcal/mol/nm^2, θ in $^\circ$, and k_A in kcal/mol) for the considered flexible models and mapping $M = 4$. The dipole moment μ is given in D.

model	σ	ϵ	σ	e	fixed<br>l	k_B	μ	
2SF	4.60 - 5.00	0.30 - 1.15	0.0 - 0.1	0.5452	2.4337	34.8621	6.3730	
**model**	**σ**	**ϵ**	**σ**	**e**	**fixed<br>l**	**θ**	**k_A**	**μ**
3SF	4.60 - 5.00	0.30 - 1.15	0.0 - 0.1	0.6493	1.7219	29.3119	9.2364	5.1953
**model**	**σ**	**ϵ**	**σ**	**e**	**fixed<br>l**	**θ**	**k_A**	**μ**
*3S*F*	4.60 - 5.00	0.30 - 1.15	0.0 - 0.1	0.5495	1.4730	87.0474	7.8318	5.3524

Table S9: Model parameters (σ in Å, ϵ in kcal/mol) with maximum likelihood for the considered models and mappings M.

M	*LJ*<br>σ	ϵ
1	2.9534	1.0140
3	4.3353	1.1826
4	4.7360	1.2094
5	5.0505	1.2369
6	5.3938	1.2056

M	*2S*<br>σ	ϵ	3S<br>σ	ϵ	3S*<br>σ	ϵ
3	4.4079	0.9329	4.3953	1.0089	4.4309	0.9398
4	4.8604	0.9522	4.8374	1.0255	4.8759	0.9128
5	5.2117	0.9996	5.2111	1.0244	5.2280	1.0223
6	5.5137	1.0496	5.5475	0.9468	5.5700	1.0464
12	7.0086	1.0068	6.9733	1.0452	7.1020	0.9948

M	*2SF*<br>σ	ϵ	3SF<br>σ	ϵ	3S*F<br>σ	ϵ
4	4.8967	0.9923	4.8328	1.0120	4.9210	0.8871

6 Maximum integration timestep

Table S10: Maximum integration timestep (in fs) for the considered models and mappings M.

M/model	*LJ*	*2S*	*2SF*	*3S*	*3SF*	*3S**	*3S*F*
1	10	/	/	/	/	/	/
3	26	22	/	2	/	20	/
4	34	28	22	8	6	26	28
5	38	34	/	12	/	32	/
6	48	40	/	18	/	36	/
12	/	70	/	66	/	66	/

7 Information about hyper-parameter models for hierarchical inference

Table S11: Parameters of the prior for the hyper-parameters for the uniform and the gamma prior models.

	$\mathcal{M}=\mathcal{U}$	$\mathcal{M}=\Gamma$
$[a_1^{\mathcal{M}}, b_1^{\mathcal{M}}]$	[0.0, 8.0]	[3.0, 6.0]
$[a_2^{\mathcal{M}}, b_2^{\mathcal{M}}]$	[0.0, 5.0]	[0.0, 1.0]
$[a_3^{\mathcal{M}}, b_3^{\mathcal{M}}]$	[0.0, 5.0]	[0.1, 5.0]
$[a_4^{\mathcal{M}}, b_4^{\mathcal{M}}]$	[0.0, 5.0]	[0.0, 1.0]
$[a_5^{\mathcal{M}}, b_5^{\mathcal{M}}]$	[0.0, 5.0]	[0.1, 2.0]
$[a_6^{\mathcal{M}}, b_6^{\mathcal{M}}]$	[0.0, 1.0]	[0.0, 3.0]

We assume that,

$$p(\boldsymbol{\varphi} \mid \boldsymbol{\psi}, \mathcal{M}) = \prod_{j=1}^{3} p(\varphi_j \mid \boldsymbol{\psi}, \mathcal{M}), \tag{S16}$$

and we consider the following two models:

1) uniform: $p(\varphi_j \mid \boldsymbol{\psi}, \mathcal{M}) = \mathcal{U}(\varphi_j \mid \psi_{2j-1}, \psi_{2j-1} + \psi_{2j})$, where $\mathcal{U}(\cdot|a, b)$ is the uniform distribution with parameters a, b and $\mathcal{M}$ is set to $\mathcal{U}$,

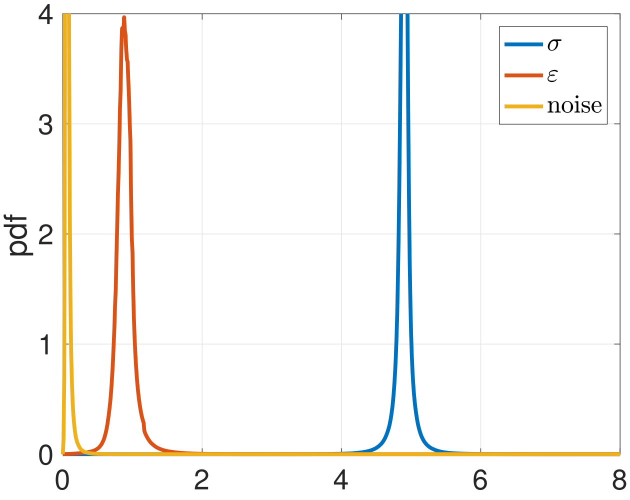

Figure S2: $p(\boldsymbol{\varphi}^{\text{new}} \,|\, \vec{\boldsymbol{d}}, \mathcal{U})$.

2) gamma: $p(\varphi_j \,|\, \boldsymbol{\psi}, \mathcal{M}) = \Gamma(\varphi_j \,|\, \psi_{2j-1}, \psi_{2j})$, where $\Gamma(\cdot|\mu, \sigma)$ is a re-parametrized gamma distribution with mean μ and standard deviation σ and $\mathcal{M}$ is set to Γ. Given μ and σ the shape and the scale of the gamma distribution are given by $k = \frac{\mu^2}{\sigma}$ and $\theta = \frac{\sigma}{\mu}$.

The prior distribution on the hyper-parameters is modeled as independent uniform,

$$p(\boldsymbol{\psi} \,|\, \mathcal{M}) = \prod_{j=1}^{6} \mathcal{U}(\psi_j \,|\, a_j^{\mathcal{M}}, b_j^{\mathcal{M}}) \,, \tag{S17}$$

where $\mathcal{M} \in \{\mathcal{U}, \Gamma\}$ and the constants $a_j^{\mathcal{M}}, b_j^{\mathcal{M}}$ are given in Table S11. The model $\mathcal{U}$ is according to the Bayesian model selection criterion, an order of magnitude more plausible and thus will be used for the further inference.

Table S12: Logarithm of the evidence for the uniform and the gamma prior models.

	$\mathcal{M} = \mathcal{U}$	$\mathcal{M} = \Gamma$
3S2	-95.1113	-98.2459
3S	-98.1956	-98.6998
2SF	-99.9770	-99.1716

References

(1) Plimpton, S. *J Comput Phys.* **1995**, *117*, 1–19.

(2) Ching, J.; Chen, Y. *Journal of engineering mechanics* **2007**, *133*, 816–832.

(3) Calderhead, B.; Girolami, M. *Computational Statistics and Data Analysis* **2009**, *53*, 4028 – 4045.

(4) Forrester A., K. A., Sobester A. *Engineering Design via Surrogate Modelling: A Practical Guide*; Wiley, 2008.

(5) Bishop, C. M. *Neural Networks for Pattern Recognition*; Oxford University Press, 1996.

(6) Wu, S.; Angelikopoulos, P.; Tauriello, G.; Papadimitriou, C.; Koumoutsakos, P. *J. Chem. Phys.* **2016**, *145*, 244112.

(7) Lide, D. R. *CRC Handbook of Chemistry and Physics*; CRC Press LLC, 2004.

(8) Kell, G. S. *J. Chem. Eng. Data* **1967**, *12*, 66–69.

(9) Marrink, S. J.; de Vries, A. H.; Mark, A. E. *J. Phys. Chem. B* **2004**, *108*, 750–760.

(10) Yesylevskyy, S. O.; Schäfer, L. V.; Sengupta, D.; Marrink, S. J. *PLoS Comput. Biol.* **2010**, *6*, e1000810.

(11) Wu, Z.; Cui, Q.; Yethiraj, A. *J. Phys. Chem. B* **2010**, *114*, 10524–10529.

(12) Braun, D.; Boresch, S.; Steinhauser, O. *J. Chem. Phys.* **2014**, *140*, 064107.

(13) Riniker, S.; van Gunsteren, W. F. *J. Chem. Phys.* **2011**, *134*, 084110.
